# Fibroblast expression of neurotransmitter receptor HTR2A associates with inflammation in rheumatoid arthritis joint

**DOI:** 10.1007/s10238-024-01352-w

**Published:** 2024-04-25

**Authors:** Chunyan Xiang, Soon-Min Hong, Bingjiao Zhao, Hui Pi, Fang Du, Xingyu Lu, Yuanjia Tang, Nan Shen, Chunxi Yang, Runci Wang

**Affiliations:** 1 Shanghai Institute of Rheumatology, Renji Hospital, School of Medicine, Shanghai Jiao Tong University (SJTUSM), Shanghai, China; 2 Department of Orthodontics, Shanghai Stomatological Hospital & School of Stomatology, Fudan University, Shanghai, China; 3 Jiangxi Provincial People’s Hospital, The First Affiliated Hospital of Nanchang Medical College, Nanchang, China; 4 Department of Rheumatology, Renji Hospital, School of Medicine, Shanghai Jiao Tong University (SJTUSM), Shanghai, China; 5 Department of Endocrinology, Xinhua Hospital, School of Medicine, Shanghai Jiao Tong University (SJTUSM), Shanghai, China; 6 Department of Orthopedics, Renji Hospital, School of Medicine, Shanghai Jiao Tong University (SJTUSM), Shanghai, China

**Keywords:** Neuroimmune, Neurotransmitter receptors, Rheumatoid arthritis, Extracellular vesicles

## Abstract

The study of neuroimmune crosstalk and the involvement of neurotransmitters in inflammation and bone health has illustrated their significance in joint-related conditions. One important mode of cell-to-cell communication in the synovial fluid (SF) is through extracellular vesicles (EVs) carrying microRNAs (miRNAs). The role of neurotransmitter receptors in the pathogenesis of inflammatory joint diseases, and whether there are specific miRNAs regulating differentially expressed HTR2A, contributing to the inflammatory processes and bone metabolism is unclear. Expression of neurotransmitter receptors and their correlated inflammatory molecules were identified in rheumatoid arthritis (RA) and osteoarthritis (OA) synovium from a scRNA-seq dataset. Immunohistochemistry staining of synovial tissue (ST) from RA and OA patients was performed for validation. Expression of miRNAs targeting HTR2A carried by SF EVs was screened in low- and high-grade inflammation RA from a public dataset and validated by qPCR. HTR2A reduction by target miRNAs was verified by miRNAs mimics transfection into RA fibroblasts. HTR2A was found to be highly expressed in fibroblasts derived from RA synovial tissue. Its expression showed a positive correlation with the degree of inflammation observed. 5 miRNAs targeting HTR2A were decreased in RA SF EVs compared to OA, three of which, miR-214-3p, miR-3120-5p and miR-615-3p, mainly derived from monocytes in the SF, were validated as regulators of HTR2A expression. The findings suggest that fibroblast HTR2A may play a contributory role in inflammation and the pathogenesis of RA. Additionally, targeting miRNAs that act upon HTR2A could present novel therapeutic strategies for alleviating inflammation in RA.

**Supplementary Information:**

The online version contains supplementary material available at 10.1007/s10238-024-01352-w.

## Introduction

Extensive evidence has highlighted the co-localization of peripheral nerve endings and immune cells within local tissues, suggesting their intricate interactions [1–3]. Neurotransmitters released by peripheral nerves are crucial in neuroimmune crosstalk, influencing the inflammatory processes and bone metabolism within joints [4–9]. Rheumatoid arthritis (RA) and osteoarthritis (OA) are two of the most common joint disorders affecting millions of people worldwide. In contrast to degenerative and non-inflammatory OA, RA is an autoimmune disease characterized by chronic inflammation of the joints leading to progressive joint damage, disability, and decreased quality of life. Whether neuroimmune crosstalk participates in joint inflammation is intriguing yet unknown.

Neurotransmitters exert their functions by binding to specific receptors. One serotonin receptor known as HTR2A is a G-protein-coupled receptor expressed on immune cells including B cells, T cells, macrophages, monocytes, dendritic cells (DCs), natural killer cells (NKs), and eosinophils [10–15], of which gene polymorphisms link to increased susceptibility to RA and other autoimmune diseases [16, 17]. Studies have shown that activation of the serotonin signaling pathway in the DSS-induced colitis mice had a pro-inflammatory effect and selective 5-HT2A receptor antagonists could lower pro-inflammatory cytokine production of macrophages [18]. In addition, selective 5-HT2A receptor antagonists inhibited antigen-specific Th1 and cytotoxic T lymphocytes (CTLs) [19]. Another study found a role of HTR2A in hypoxia-associated proliferation of pulmonary artery fibroblast [20]. However, the role of HTR2A in synovial fibroblasts, which contribute to joint damage by promoting both inflammation and bone destruction, remains poorly understood.

Extracellular vesicles are membrane-derived nanoscale particles, containing bioactive molecules like RNAs, proteins, and lipids, released by cells into extracellular space to facilitate cell–cell communication [21]. MicroRNAs are 18 ~ 25 nucleotide non-coding RNAs. Changes in their expression indicate early responses triggered by external stimuli because miRNA amounts in cells can change rapidly [22]. A growing body of evidence illustrated that a single miRNA can regulate over 200 genes, and conversely, multiple miRNAs can collaboratively regulate one gene [23, 24]. MiRNAs are well studied to target 3′ untranslated regions (3′ UTRs) and non-3' UTR regions in mRNA transctipts [25], thereby regulating protein synthesis. This dynamic but complicated interaction between miRNAs and their target genes plays an important role in diseases. For example, miR-34a in bone marrow mesenchymal stem cell (BM-MSC)-derived EVs could reduce RA inflammation by inhibiting the cyclin I/ATM/ATR/p53 signaling pathway [26]. miR-103a contained in macrophage-derived EVs could exacerbate RA by inhibiting the expression of HNF4A to activate the JAK/STAT3 signaling pathway [27]. However, it remains unclear whether specific miRNAs regulate neurotransmitter receptors (NTRs) within joints, contributing to the distinct inflammatory processes and bone metabolism in RA and OA.

In this study, we aim to comprehensively examine the neurotransmitter receptor expression profile in arthritis joints and explore the possible regulation mechanisms via miRNAs in extracellular vesicles to pathogenic neurotransmitter receptors, which may offer potential therapeutic targets and methods for future RA interventions.

## Methods

### Single-cell RNA sequencing data analysis

We analyzed public-available single-cell RNA-seq data from ImmPort (study SDY998) featuring articular synovial T cells, B cells, monocytes, and fibroblasts from 3 OA and 18 RA patients, sequenced using the CEL-Seq2 method [22]. Following UMI-based gene quantification, log2(CPM) transformation, and quality control, 5265 cells and 32391 genes were used for further analysis.

### miRNA sequencing data analysis

We analyzed public-available RNA-seq of extracellular vesicles, isolated from SF of RA patients with high (n = 7)- or low (n = 5)-grade inflammation classified by leukocyte counts from Foers et al. [28]. Differentially expressed miRNAs were defined with adjusted *P*-value < 0.05 and log2FC  > 1.2 or  < − 1.2.

### miRNA target prediction

miRNA targeting HTR2A was predicted by RNAhybrid 2.2 [29] and TargetScanHuman 8.0 [30]. Overlapping results of RNAhybrid, conserved sites in results of TargetScan and miRNAs lower expressed in high-inflammation RA, we identified differentially expressed miRNAs targeting HTR2A in RA.

### Patient material

Synovial fluid and tissue were obtained from RA and OA patients with informed consent and Renji Hospital Human Research Ethics Committee approval (identification no. 2013-126). Cells and supernatant of SF were frozen at -80℃ until required. Half of each synovial tissue piece was diced, digested with 100U/ml liberase TL and 100U/ml DNase I (Roche, Switzerland) at 37 °C for 15 min, and then passed through filter. ST cells were preserved in liquid nitrogen for future experiments. The other half of each synovial tissue piece was fixed for Immunohistochemistry (IHC) staining.

### Immunohistochemistry staining and analysis

Paraffin-embedded tissue sections (5 μm) were prepared using RM2125 (Leica, Germany). After heating at 55 °C for 30 min, sections were deparaffinized with xylene, rehydrated with ethanol, and antigen retrieval at 95 °C for 20 min. Endogenous peroxide was removed with 3% H_2_O_2_ for 15 min. Slides were blocked and then stained with primary antibodies, anti-human HTR2A (Invitrogen, USA), IL-6 and CXCL12 (Proteintech, USA) overnight at 4 °C. Subsequent steps included secondary antibody staining, HRP treatment, DAB staining, and hematoxylin counterstaining.

Slides were digitally scanned (Servicebio, China) and quantified by QuPath-0.4.2 software. Fibroblast identification criteria were Nucleus Perimeter  > 19 and Nucleus Circularity  < 0.8; marker positivity was determined according to Cytoplasm DAB OD max value,  > 0.6 for HTR2A,  > 0.4 for IL-6,  > 0.7 for MMP13,  > 0.55 for MMP14 and  > 0.7 for CXCL12.

### Exosome isolation and identification

Exosome isolation was performed using differential ultracentrifugation method as described [31]. The concentration and size distribution of exosomes were identified by Nanoparticle Tracking Analysis (NTA) method via NanoFCM, China. Morphology identification of exosomes was performed with Transmission Electron Microscopy (TEM). Exosome-specific markers (CD9, CD81, CD63) were verified by western blot.

### Western blot

EVs or cells were resuspended in lysis buffer. Protein concentration was detected by BCA Kit (Beyotime BioTech, China). Purified proteins were separated in 10%SDS-PAGE and transferred to PVDF membrane at 350 mA for 50 min. After washing and blocking, the membrane was incubated overnight with human primary antibodies CD9, CD81, CD63, and Calnexin (1000 × dilution, Beyotime BioTech, China), HTR2A (1000 × dilution, Abclonal, China), GAPDH (2000 × dilution, Abclonal, China). Secondary antibodies were applied for 120 min, and images were acquired using a chemiluminescence detection system (Beyotime BioTech, China).

### Fibroblast, T cell and monocyte isolation

Single-cell suspension of synovial tissue was purified into fibroblasts after 4 passages in one month. Single-cell suspension of SF was stained with FITC-TCRb and percp5.5-CD14 (Biolegend, USA) at dark 4 °C for 10–15 min. After washing and centrifugation, cell pellets were resuspended with propidium iodide/PBS buffer (Biolegend, USA) and sorted into T cells and monocytes by BD FACSAria™ III. Single-cell suspension of each subset was pooled from 6 OA patients.

### miRNA extraction and quantification

miRNA extraction used the miRcute Serum/Plasma miRNA Isolation Kit (TIANGEN, China) as instructed. Briefly, 900 ul lysis buffer was mixed with each 200 ul EV-biofluid or each cell pellet thoroughly. 1 pmol cel-miR-39 (RIBOBIO, China) per 200 ul EV-biofluid or 10^5 cells was added as an exogenous control. The mixture was placed at room temperature for 5 min to separate nucleic acids and protein. RNA was separated into aqueous phase by 200 ul of chloroform and then transferred into twice the volume of absolute ethanol. After passing the obtained liquid through the Column miRelute, 700 ul of MRD buffer and 500 ul of RW buffer were added into the Column miRelute for RNA purification. Lastly, RNA was eluted in 20 ul of RNase-Free ddH_2_O.

MiRNAs were reverse transcribed and the expression levels were measured by quantitative real-time polymerase chain reaction (qPCR) on a QuantStudio 7 Flex instrument (Applied Biosystems). MiRNA PCR was performed with the Bulge-Loop miRNA qRT-PCR Starter Kit, Bulge-Loop hsa-miR-23b-3p Primer Set, Bulge-Loop hsa-miR-23b-5p Primer Set, Bulge-Loop hsa-miR-214-3p Primer Set, Bulge-Loop hsa-miR-3120-5p Primer Set, Bulge-Loop hsa-miR-615-3p Primer Set and Bulge-Loop cel-miR-39 Primer Set (RIOBO, China) according to manufacturer’s instructions. cDNA synthesis reaction contained 1 μL of total RNA, 0.5 μL of Bulge-LoopTM miRNA RT Primer (5 μM), 1 μL of RTase Mix, 1 μL 5 × Reverse Transcription Buffer, 1.5 μL of RNase-free H_2_O. The reaction mixture was incubated at 42 °C for 60 min, followed by 70 °C for 10 min. The synthesized cDNA was stored at  − 20 °C until further use. qPCR reactions were performed in a total volume of 10 μL, containing 5 μL of 2 × SYBR Green Mix, 1 μL of template DNA, 0.4 μL of Bulge-LoopTM miRNA Forward Primer (5 μM) and 0.4 μL of Bulge-LoopTM Reverese Primer (5 μM) and 3.2 μL of RNase-free H_2_O. The qPCR cycling conditions consisted of an initial denaturation step at 95 °C for 10 min, followed by 40 cycles of denaturation at 95 °C for 2 s, annealing at 60 °C for 20 s, and extension at 70 °C for 10 s. A final extension step was performed at 72 °C for 5 min. Assays set up in 96-well PCR plates (Integrated Sciences, Australia) included 3 duplicates of each sample.

For miRNAs extracted from SF-EVs, data was normalized by the initial synovial fluid volume which was used for EV isolation, and was calibrated by the cel-miR-39 to eliminate the bias caused by different synovial fluid volume, RNA isolation efficiencies and PCR efficiencies among samples. For miRNAs extracted from cells, data was normalized by exogenous control. Gene expression was relatively quantified with 2^−ΔΔCt^ method.

### miRNA transfection

RA fibroblast cell line (PriMed-Icell-003, icell bioscience, China) at 60–80% confluence was transfected. Lipofectamine® RNAiMAX Reagent (Thermo Fisher Scientific, USA) was diluted in Opti-MEM® Medium (Gibco, USA) and mixed with miRNA mimics (RIBOBIO, China) in Opti-MEM® Medium at a 1:1 ratio. After 5 min at room temperature, the miRNA-lipid complex was added into 6 wells plate to incubate cells at 37℃ for 2–3 days. miRNAs mimics include hsa-miR-23b-3p, hsa-miR-23b-5p, hsa-miR-214-3p, hsa-miR-3120-5p, hsa-miR-615-3p. Cel-miR-39 acts as a negative control to check the off-target effect. Also, blank control was designed to assess any non-specific effects or variations in gene expression resulting from factors such as transfection reagents, culture conditions, or sample processing techniques.

### Statistics

Analyses were performed using R 4.2.0 and GraphPad Prism 8.0. Data was analyzed by Student t-test, Mann–Whitney test, Chi-square test, Fisher’s exact test and Spearman’s correlation when applicable. Differences were considered significant if *p* < 0.05 (indicated as * for *p* < 0.05, ** for *p* < 0.01, *** for *p* < 0.001 and **** for *p* < 0.0001 when measurement data, and * for *p* < 0.05 when enumeration data).

## Results

### Identification of differentially expressed neurotransmitter receptors in arthritis joints

**Fig.1:**
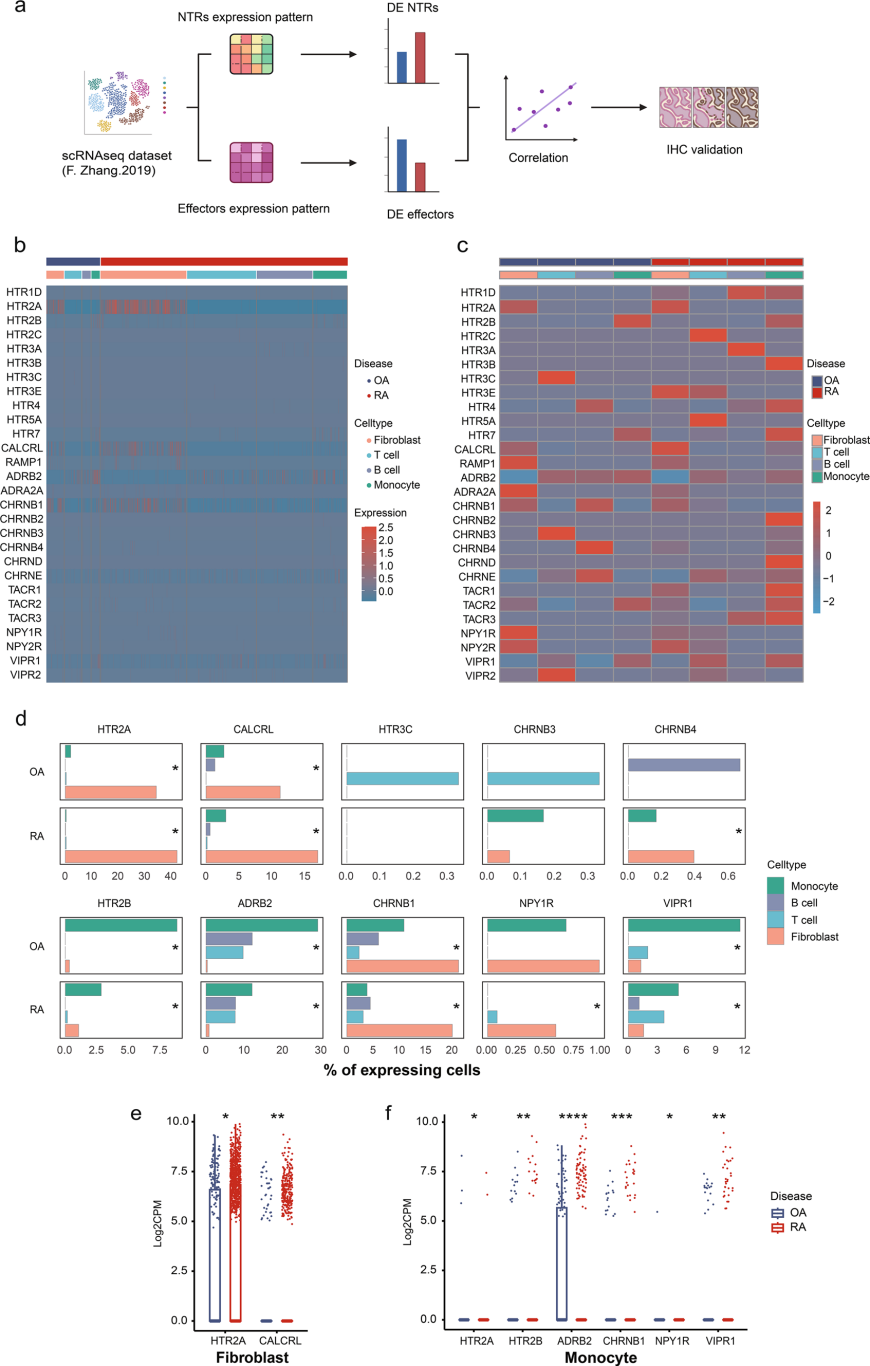
Neurotransmitter receptors (NTRs) expression pattern in joint synovial tissue of RA and OA patients. **a** Workflow for target genes screening and validation. Created with BioRender.com. **b** NTRs expression in each cell of RA and OA. **c** NTRs average expression pattern in different cell types of RA and OA. **d** Percent of differentially expressed NTRs (DE NTRs) in different cell types, statistical test by Chi-square or Fisher’s exact test, *p < 0.05. Log2CPM of DE NTRs in Fibroblast (**e**) or Monocyte (**f**) comparing RA with OA using Mann–Whitney U test, *p < 0.05, **p < 0.01, ***p < 0.001, ****p < 0.0001

We analyzed a public dataset of scRNA-seq performed on synovial tissue from RA and OA patients [32]. 5265 cells were examined, including 1844 fibroblasts, 1529 T cells, 1142 B cells and 750 monocytes. Expression patterns of known NTRs were mapped out on each type of cells from the joints (Tables S1, S2). Differentially expressed NTRs were identified in specific cell types. A similar approach was used to identify differentially expressed effector molecules (Tables S3, S4). Correlation among the NTRs and effector molecules was analyzed (Tables S5, S6). To validated these expression patterns, immunohistochemistry staining of RA and OA joints was performed. This study approach was summarized in Fig. 1a.

We examined the pattern of NTR expression on each cell (Fig. 1b), the average expression of NTRs on different cell types (Fig. 1c), and the abundance of these NTRs expressed on different cell types (Figs. 1d and S1a). Among all the NTRs examined, HTR2A, CALCRL and CHRNB1 were mostly expressed by fibroblasts compared to leukocytes both in RA and OA. HTR2A was expressed on 34.3% and 42.1% of fibroblasts in OA and RA, CALCRL was expressed on 11.2% and 16.9% respectively, and CHRNB1 was expressed on approximately 20% of both OA and RA fibroblasts. These NTRs were enriched in fibroblasts and only expressed by less than 10% of leukocytes. In contrast, ADRB2 was barely expressed by fibroblast, while it was the most expressed NTR by leukocytes, including 12% of monocytes in RA and 29.1% of monocytes in OA, and nearly 10% of T and B cells (Figs. 1d and S1a). Besides higher percentage of expression, HTR2A and CALCRL expression on each cell measured by log2CPM are increased in RA fibroblasts(Fig. 1e). Monocytes in RA and OA showed different expression of many NTRs, including ADRB2, CHRNB1, VIPR1, HTR2B, NPY1R and HTR2A(Fig. 1f), however, except for ADRB2 and VIPR1(5.2% in RA and 11.5% in OA), the remaining NTRs were expressed on very few monocytes (Fig. 1d, f). Different but small amount of cells expressed of HTR3C and CHRNB3 on RA and OA T cells, and CHRNB4 on RA and OA B cells was also identified (Fig. S1b, c). The expression level of other NTRs were not significantly different on these cell types (Fig. S1d–g).

The above findings identified HTR2A as the most abundant NTR on fibroblast, and it’s significantly upregulated in the inflammatory RA joints. To further explore whether the difference of HTR2A in RA and OA is associated with the inflammatory microenvironment, we examined the expression of inflammatory effector molecules in these joints.

### Upregulated HTR2A correlate with inflammation in arthritis joints

**Fig. 2:**
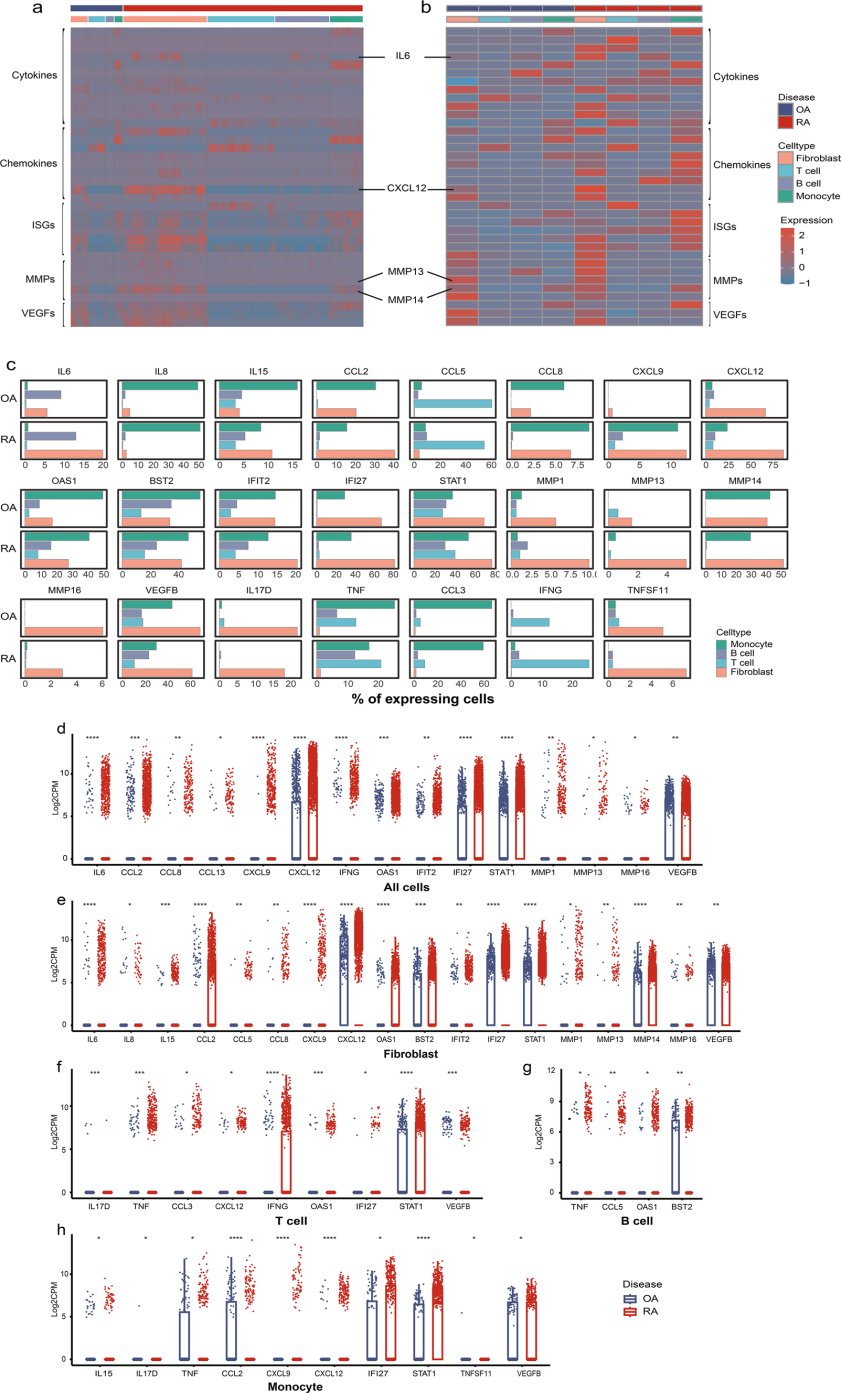
Effectors expression pattern in joint synovial tissue of RA and OA patients. **a** Effectors expression in each cell of RA and OA. **b** Effectors average expression pattern in different cell types of RA and OA. **c** Percent of differentially expressed effectors (DE effectors) in different cell types. **d**–**h** Log2CPM of DE effectors in all cells or each cell type comparing RA with OA using Mann–Whitney U test, *p < 0.05, **p < 0.01, ***p < 0.001, ****p < 0.0001. ISGs, IFN stimulated genes

To examine the pattern of several types of inflammatory effector molecules involved in the pathology of arthritis, including cytokines and molecules in their downstream inflammatory pathways, chemokines, and molecules related to joint erosion and angiogenesis, we looked at their expression level of each cell (Fig. 2a), averaged expression on different cell types (Fig. 2b), and the expression percentage on different cell types (Fig. 2c and S2a).

Compared to non-inflammatory OA, higher levels of IL-6, IFN-γ, IFN stimulated genes (OAS1, IFIT2, IFI27 and STAT1), chemokines including CCL2, CCL8, CCL13, CXCL9, and CXCL12, and MMP1, MMP13, MMP16 and VEGFB were observed on all cell types in RA joint (Figs. 2d and S2b), indicating a highly inflammatory microenvironment in RA. Higher percentage of fibroblasts in RA expressed IL-6, CCL2, CXCL12, IFIT2, IFI27, MMP1, and MMP14, over other cell types in RA and OA (Figs. 2c and S2a). In addition, fibroblast in RA showed a higher level of these effectors than fibroblast in OA(Figs. 2e and S2c). More percentage of RA T cells expressed higher IFNG, STAT1 and TNF, whereas RA B cells displayed higher TNF, CCL5 and OAS1 (Figs. 2c, f, g and S2a, d, e). Many molecules were enriched on more than 20% monocytes(Figs. 2c and S2a), but not differentially upregulated in RA compared to OA except for TNF, CCL2, IFI27, STAT1 and VEGFB (Figs. 2h and S2f).

**Fig. 3:**
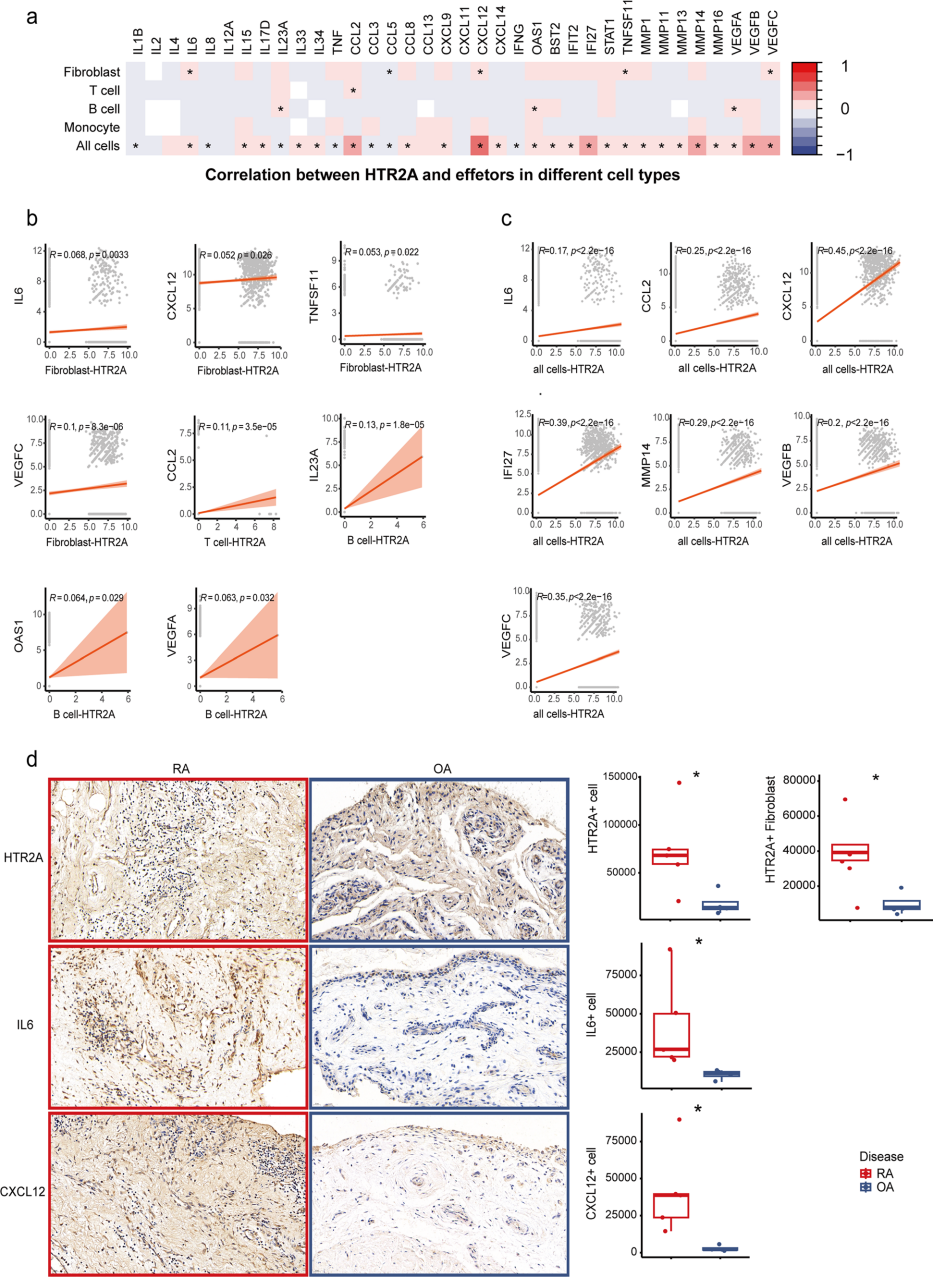
Correlation between HTR2A and inflammatory effectors. **a** Correlation heatmap between HTR2A and effectors in different cell types or all cells using Spearman’s correlation. Color key represents Spearman correlation values (r), * represents *p* < 0.05. **b**, **c** Statistically significant correlation plot between HTR2A and effector molecules in different cell types or all cells. **d** 40 × field view and quantification of immunohistochemical staining of HTR2A and significant inflammation effectors IL6, CXCL12 in joint synovial tissue of RA (n = 5) and OA (n = 4) patients, *p* values were calculated by Mann–Whitney U test, *p < 0.05, **p < 0.01, ***p < 0.001, ****p < 0.0001

To investigate the relationship between upregulated HTR2A in RA and the aforementioned inflammatory effectors in the microenvironment, we conducted a correlation analysis. We found the expression of HTR2A positively correlated with IL6, CCL5, CXCL12, TNFSF11 and VEGFC in fibroblast. We also found various positive correlations between HTR2A and inflammatory effector molecules across all cells, with the most robust correlation observed between HTR2A and CXCL12 (R = 0.45) (Fig. 3a–c).

IHC staining confirmed upregulated expression of HTR2A on RA whole ST level and fibroblast level(Fig. 3d). Increased expression of IL-6 and CXCL12 (Fig. 3d) were also found in RA ST, while MMP13 and MMP14 showed inconsistent expression pattern with the scRNA-seq data (Fig. S3a, b).

These findings identified and confirmed the elevated HTR2A in inflammatory RA joints. To investigate what could convey the inflammatory environmental cues and regulate the different HTR2A expression in RA and OA, we next examined extracellular vesicles from RA and OA synovial fluid.

### miRNAs targeting HTR2A are differentially enriched in RA and OA synovial fluid, and mostly derived from monocytes.

**Fig. 4:**
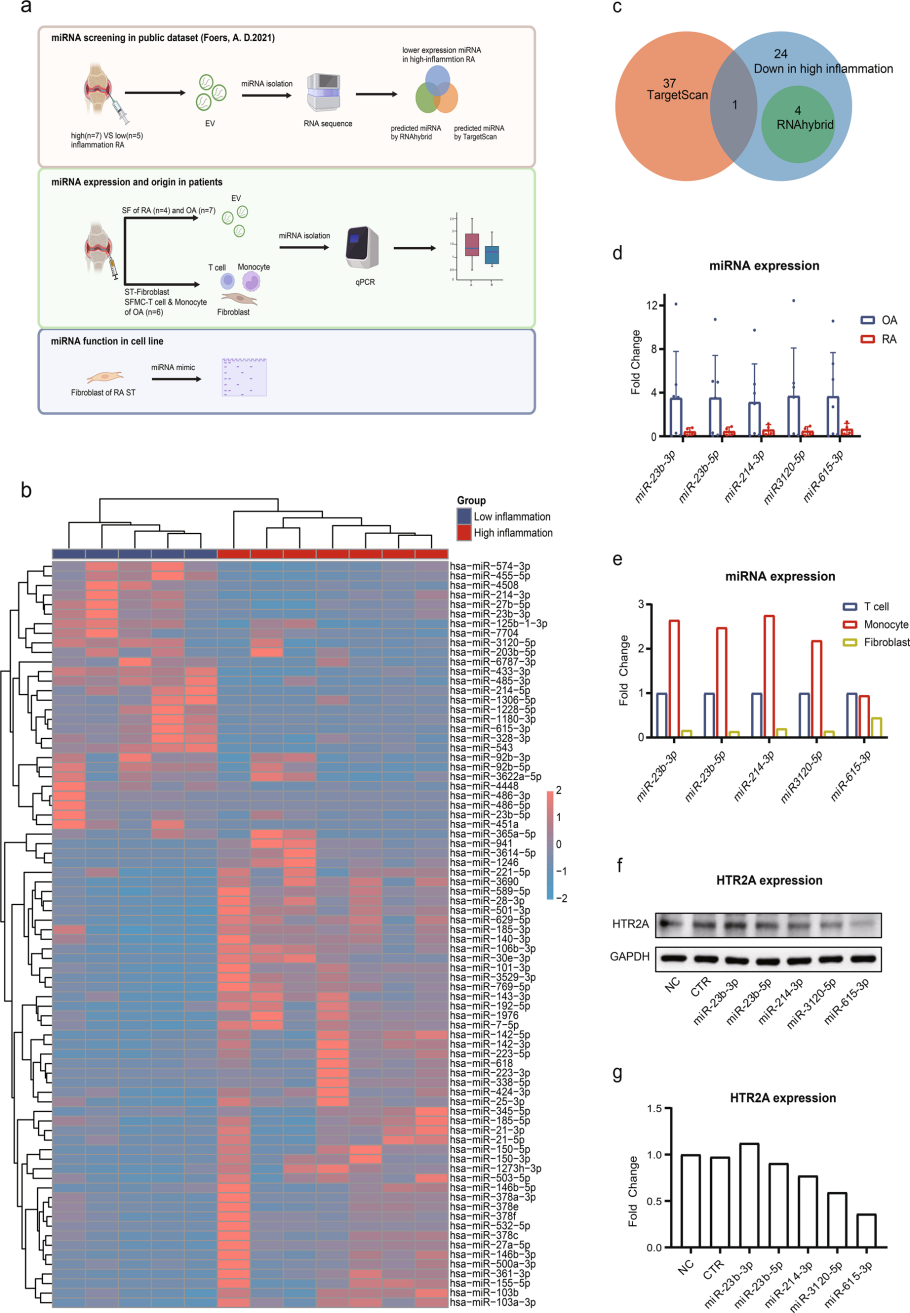
Identification of differentially expressed miRNAs targeting HTR2A. **a** Workflow of HTR2A targeted miRNAs screening and validation. Created with BioRender.com. **b** Heatmap of miRNAs expression pattern in extracellular vesicles isolated from joint synovial fluid of RA patients with high (n = 7) or low (n = 5) grade inflammation classified by leukocyte counts. **c** Venn diagram shows miRNAs targeting HTR2A predicted by TargetScan and RNAhybrid databases, and miRNAs lowly expressed in the high-inflammation RA group. The expression of miRNAs targeting HTR2A, isolated from SF EVs (**d**) or SF T cells, SF monocytes and synovial tissue fibroblasts (**e**) were tested by qPCR. *p* values were calculated by t-test. *p < 0.05, **p < 0.01, ***p < 0.001. **f**, **g** Western blot analysis and quantification of HTR2A in fibroblast after miRNAs transfection. NC, cel-miR-39 as negative control; CTR, blank control

We analyzed a public dataset of RNA-seq performed on SF EVs. Among the 78 differentially expressed miRNAs (Table S7), 49 were elevated and 29 were decreased in high-inflammation joints compared with low-inflammation joints. (Fig. 4b). Five miRNAs, miRNA-23b-3p, miR-23b-5p, miR-214-3p, miR-615-3p and miR-3120-5p, could regulate HTR2A expression identified by intersecting RNAhybrid hits (Table S8), TargetScan hits (Table S9) and miRNAs lowly expressed in high-inflammtion joints (Fig. 4c). Their sequences binding to HTR2A were predicted (Fig. S4a).

The expression of 5 miRNAs was validated by qPCR isolating EVs from RA (n = 4) and OA (n = 7) SF. EVs quality was confirmed using NTA, TEM, and western blot (Fig. S4b–d). Compared to OA, 5 miRNAs had a lower expression trend in RA SF EVs (Fig. 4d).

The source of target miRNAs was examined by sorting T cells, monocytes from OA SF and fibroblasts from OA ST (n = 6). miRNA-23b-3p, miR-23b-5p, miR-214-3p, miR-3120-5p were mainly derived from monocytes while miR-615-3p was mainly came from both in T cells and monocytes (Fig. 4e). By transfecting miRNAs mimics into RA fibroblasts, miR-214-3p, miR-3120-5p and miR-615-3p were verified to repress fibroblast HTR2A expression (Fig. 4f, g).

All in all, we found elevated HTR2A on fibroblasts associated with inflammation in RA joints. miR-214-3p, miR-3120-5p and miR-615-3p, decreased in RA synovial fluid EVs compared with osteoarthritis, were validated to regulate HTR2A expression and mostly released from monocytes.

## Discussion

Neuroimmune crosstalk is integral to the pathophysiology of immune diseases, with various cell types expressing distinct NTR patterns that influence their function [9]. In this study, we systemically examined NTR expression in arthritis joints and found increased expression of serotonin receptor HTR2A on RA synovial fibroblast.

Increased serotonin in RA serum and synovial fluid has been reported [10], with activated platelets as a source [33]. Serotonin signaling promotes pro-inflammatory Th17 cell differentiation and inflammatory cytokines production in RA mice model [34], implicating serotonin contributes to the inflammatory process in arthritis by regulating immune cell functions. Here we demonstrated serotonin receptor HTR2A enriched in synovial fibroblast compared to other immune cells, and it was elevated in the inflammatory condition. This finding reinforces the important role of serotonin and its receptor HTR2A in RA inflammation and adds another potential mechanism by which serotonin works besides acting on immune cells.

Besides its role in inflammation, serotonin is closely related to bone metabolism and pain perception. High-level serotonin negatively predicts bone mineral density in RA for suppressing osteoblasts [34] and use of SSRIs is related to a higher risk of fractures in RA patients [35]. Serotonin, in combination with other inflammatory mediators, contributes to hyperalgesia by exciting and sensitizing afferent nerve fibers in peripheral inflammatory microenvironment [36]. Blocking HTR2A at the site of inflammation inhibited activation of spinal dorsal horn neurons in rats [37], indicating targeting HTR2A in the periphery can be a promising therapy for relieving chronic inflammatory pain. In this experiment, we did not find a correlation between HTR2A and MMPs, but whether HTR2A participates in bone metabolism through other pathways needs further study. Whether and how HTR2A may be involved in RA joint chronic pain is an interesting question.

Genetic factors and epigenetic abnormalities are both important contributors to RA. Genetic variants and methylation alterations in HTR2A have been shown to play a significant role in RA. There is a significantly lower frequency of rs6313 TT genotype in RA patients over controls, and the frequency of TCTT combinations is considerably lower while the frequency of CTCC combinations is significantly higher in RA patients [38]. T cells from RA patients with TC haplotype produce higher levels of TNF-α, IL-6, and IFN-γ, and monocytes have higher levels of TNF-α in response to LPS stimulation [39]. Besides, the interaction between a protective haplotype in HTR2A and HLA-DRB1 SE alleles correlates with the risk of RA autoantibody positivity [16]. Hypermethylation of promoter region of HTR2A has been discovered in peripheral blood of RA patients compared to OA and healthy control, which is associated with inflammation and disease activity [40]. Apart from DNA methylation, miRNAs regulate gene expression epigenetically via mRNA degradation and post-translational regulation [41]. For the most studied mechanism, miRNAs bind to the 3' UTR of their target mRNA to induce translational repression and mRNA deadenylation and decapping, thus leading to mRNA degradation. miRNA binding sites have also been detected in 5′ UTR, coding sequence, and promoter regions. For miRNAs binding to non-3' UTR regions, post-translational alteration rather than mRNA degradation is proposed as their mechanism of action, affecting protein level expression rather than the mRNA level of the target gene [42]. Among the miRNAs we found, miR-214-3p and miR-3120-5p are predicted to bind the CDS region of HTR2A and miR-615-3p is predicted to bind the 3'-UTR region of HTR2A. These three miRNAs could decrease the expression of HTR2A on fibroblasts. Their lower expression in RA synovial fluid-derived EVs over OA partially accounts for the higher HTR2A expression in inflammatory joints.

In addition to regulating HTR2A on fibroblast, these miRNAs also contribute to arthritis in other means. For example, exosomal miR-214 released by osteoclasts could inhibit the function of osteoclasts in RA [43], miR-3120 is found to increase the killing effect on virus by affecting the expression of STAT and IRF, promoting the expression of cytokines, such as interleukins and chemokines [44], and miR-615-3p participates in the development and progression of osteoarthritis by promoting inflammatory cytokines including IL-1, IL-6, IL-α, and inhibiting chondrogenic differentiation of bone marrow mesenchymal stem cells (BMSCs) [45].

In conclusion, serotonin potentially contributes to pro-inflammatory processes, bone destruction, and pain hypersensitivity in RA joints. The key peripheral serotonin receptor HTR2A is involved. Further knowledge of these processes or HTR2A expression regulation may aid in drug development. We found the increased HTR2A on fibroblast in inflammatory joint is partly due to the decreased miRNAs targeting HTR2A carried by exosomes, which explore a new potential therapeutic target and effective therapeutic carriers for RA treatment.

Larger cohorts are required to validate these findings due to the small sample size. Whether there are other neurotransmitters participating in immune response regulation, and whether exosome could be a novel therapeutic method calls for more investigation. We provide further evidence of neuroimmune crosstalk in RA and OA. These contribute to a better understanding of pathogenesis and offer new therapeutic strategies for RA treatment.

## Supplementary Information

Below is the link to the electronic supplementary material.

Supplementary file1 (PDF 10042 KB)

Supplementary file2 (PDF 27216 KB)

Supplementary file3 (PDF 388 KB)

Supplementary file4 (PDF 9677 KB)

Supplementary file5 (DOCX 17 KB)

Supplementary file6 (XLSX 1429 KB)

## Data Availability

Single-cell RNA-seq data were downloaded from https://www.immport.org/shared/study/SDY998. MiRNA Sequencing Data were obtained at https://www.ncbi.nlm.nih.gov/pmc/articles/PMC8125513/table/ijms-22-04910-t004/. Other data generated during this study are included in this published article and its supplementary files.
