# Supplementary figures and images for: Fibroblast expression of neurotransmitter receptor HTR2A associates with inflammation in rheumatoid arthritis joint
Source: Clin Exp Med. 2024 Apr 25;24(1):84. doi: 10.1007/s10238-024-01352-w (PMC11045650; doi:10.1007/s10238-024-01352-w)

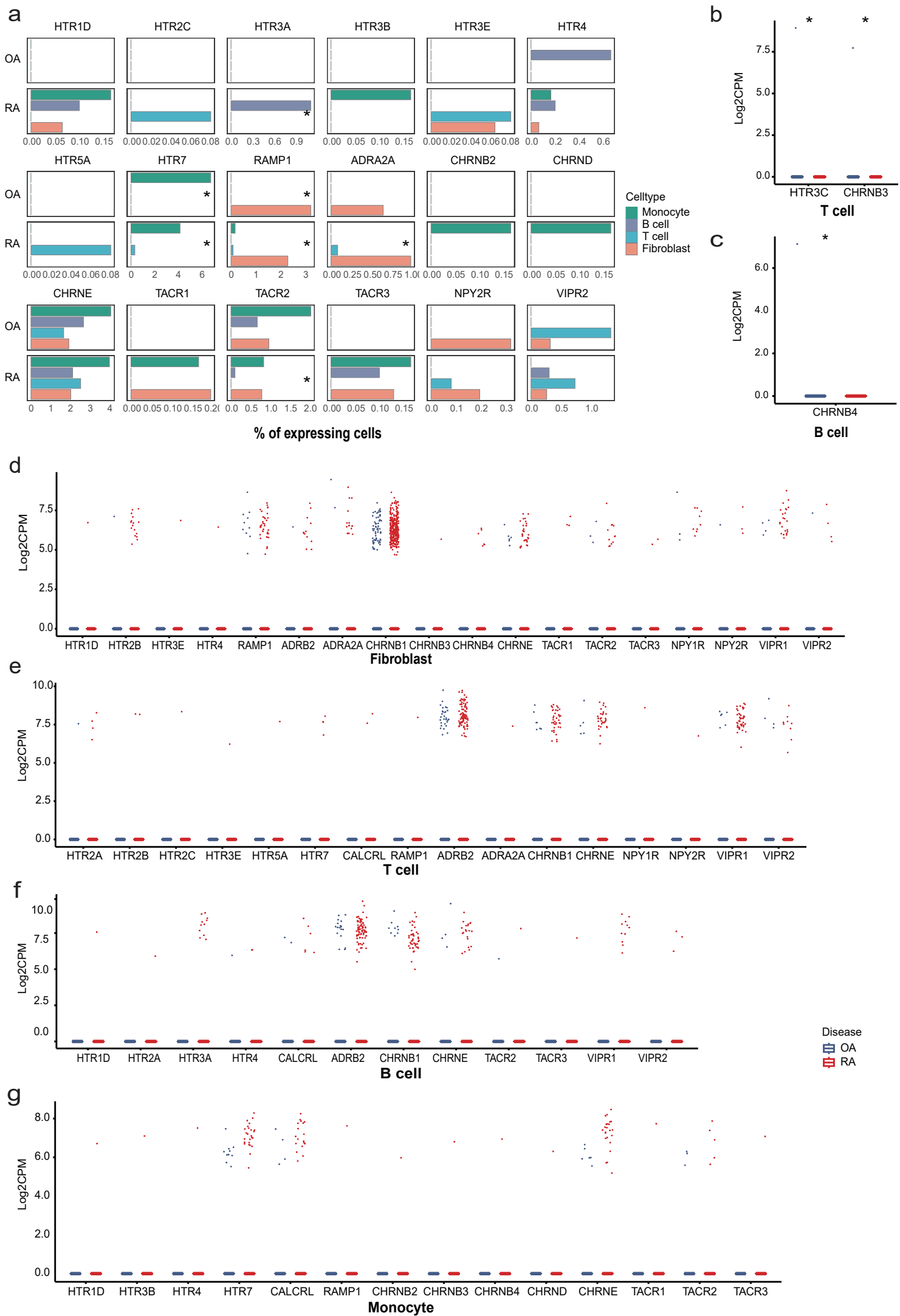

Supplement: Supplementary file 1 — Supplementary file1 (PDF 10042 KB) [file 10238_2024_1352_MOESM1_ESM.pdf]

**a**

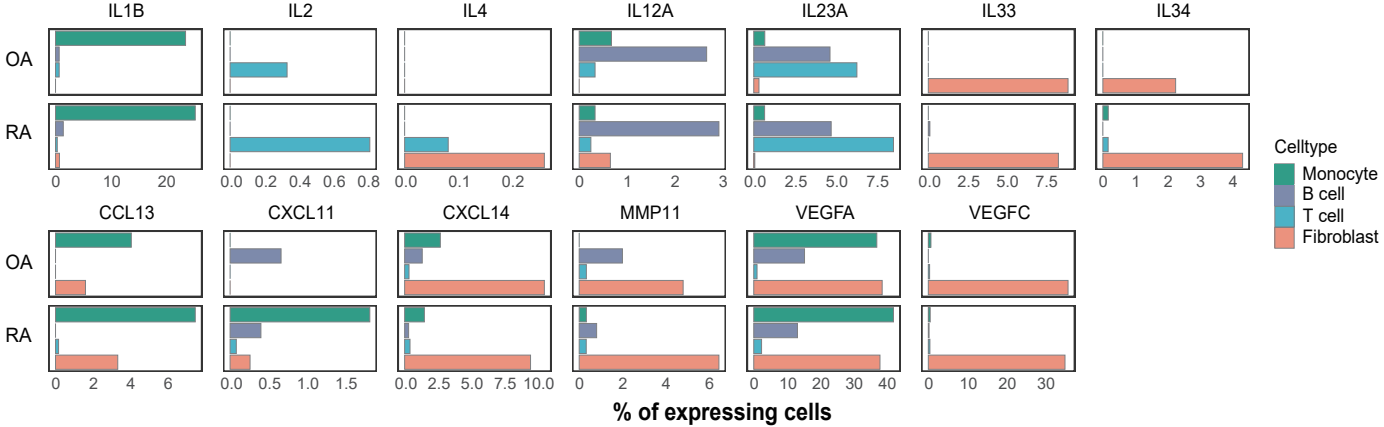

**b**

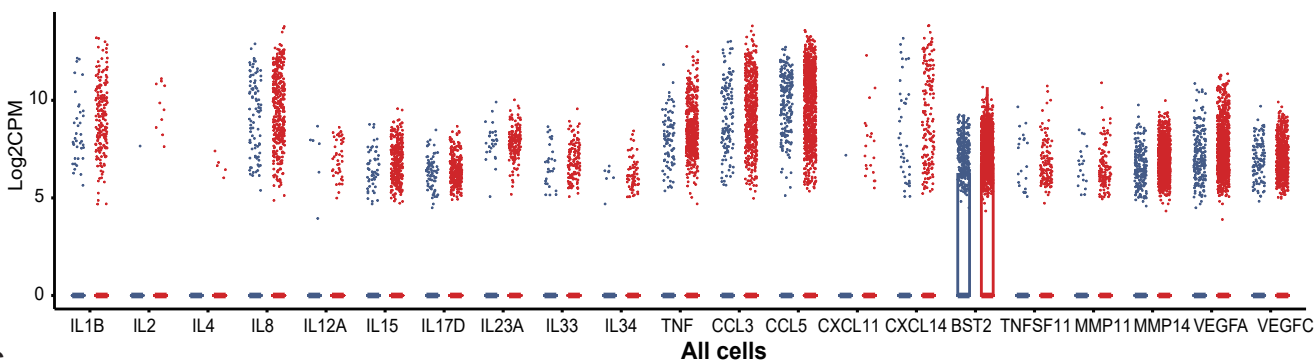

**c**

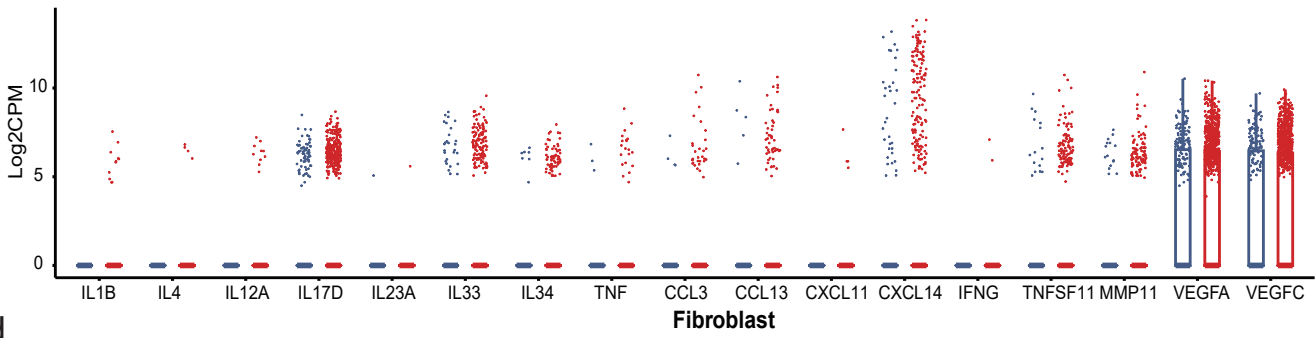

**d**

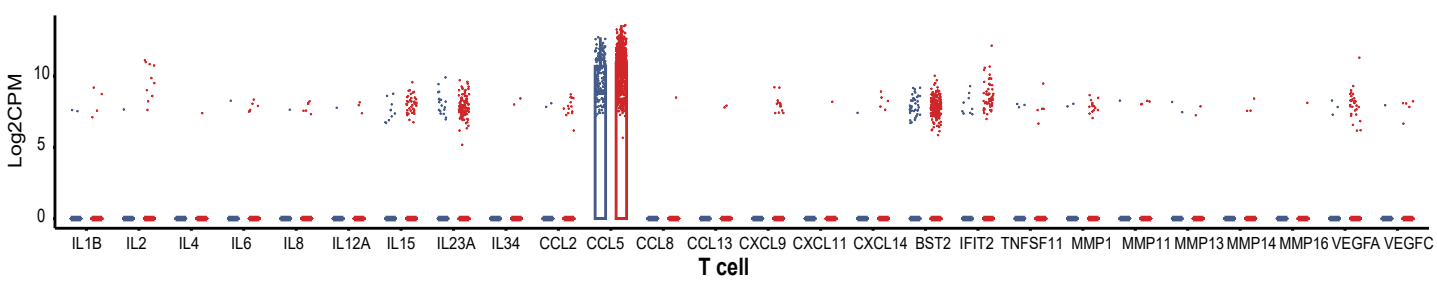

**e**

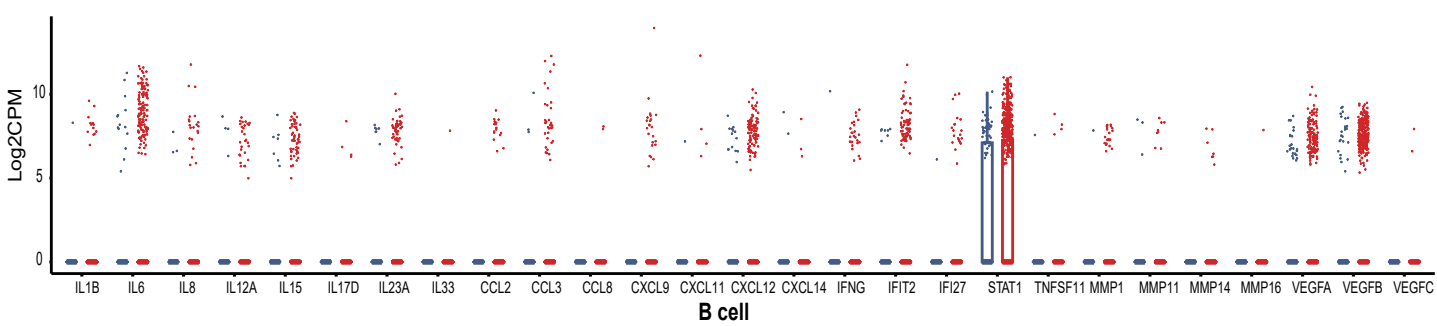

**f**

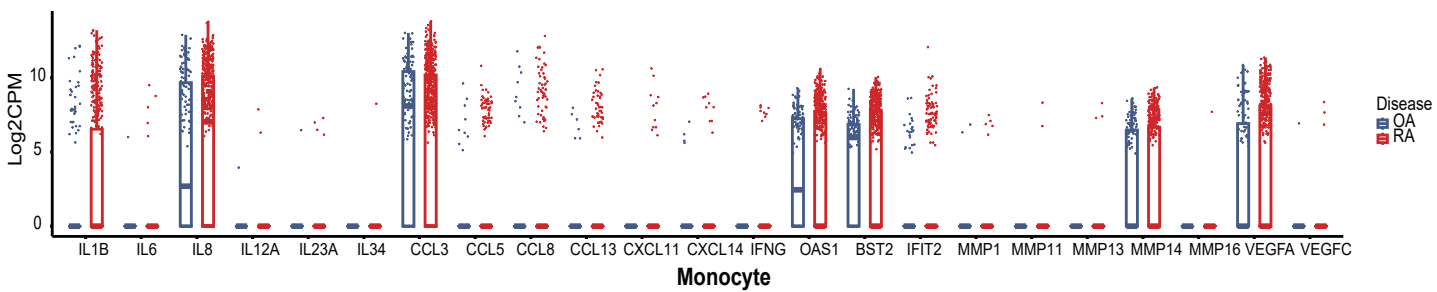

Supplement: Supplementary file 2 — Supplementary file2 (PDF 27216 KB) [file 10238_2024_1352_MOESM2_ESM.pdf]

a

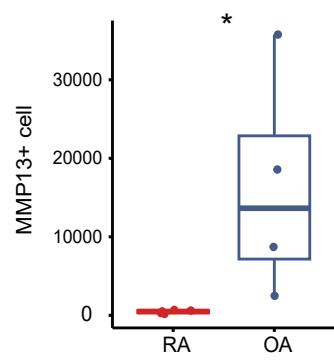

b

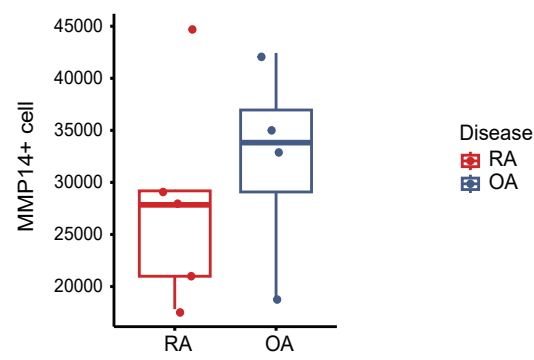

Supplement: Supplementary file 3 — Supplementary file3 (PDF 388 KB) [file 10238_2024_1352_MOESM3_ESM.pdf]

a

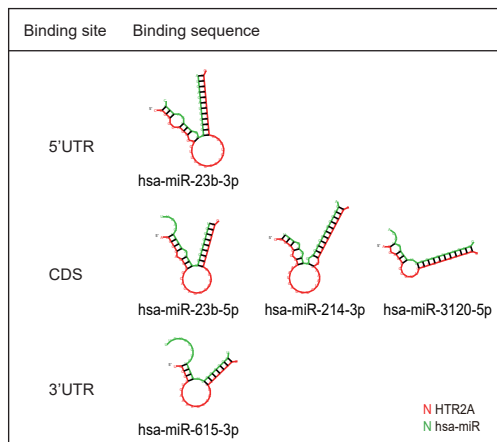

b

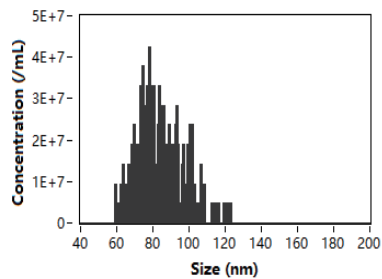

c

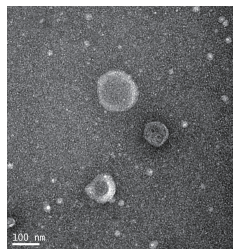

d

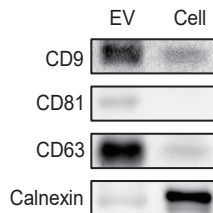

Supplement: Supplementary file 4 — Supplementary file4 (PDF 9677 KB) [file 10238_2024_1352_MOESM4_ESM.pdf]
