# Supplementary material for: Fibroblast expression of neurotransmitter receptor HTR2A associates with inflammation in rheumatoid arthritis joint
Source: Clin Exp Med. 2024 Apr 25;24(1):84. doi: 10.1007/s10238-024-01352-w (PMC11045650; doi:10.1007/s10238-024-01352-w)
Supplement: Supplementary file 5 — Supplementary file5 (DOCX 17 KB) [file 10238_2024_1352_MOESM5_ESM.docx]

Supplementary Figure 1. Neurotransmitter receptors (NTRs) expression pattern in joint synovial tissue of RA and OA patients. (a) Expressing cell percent of non-DE NTRs in different cell types, statistical test by Chi-square or Fisher's exact test, *p < 0.05. (b-c) Log2CPM of DE NTRs in T cell or B cell comparing RA with OA using Mann–Whitney U test. (d-g) Log2CPM of non-DE NTRs in each cell type comparing RA with OA using Mann–Whitney U test. *p < 0.05, **p < 0.01, ***p < 0.001，****p < 0.0001.

Supplementary Figure 2. Effectors expression pattern in joint synovial tissue of RA and OA patients. (a) Expressing cell percent of non-DE effectors in different cell types. (b-f) Log2CPM of non-DE effectors in all cells or each cell type comparing RA and OA using Mann–Whitney U test. *p < 0.05, **p < 0.01, ***p < 0.001，****p < 0.0001.

Supplementary Figure 3. IHC staining quantification of MMP13 and MMP14. (a-b) Quantification of immunohistochemical staining of MMP13 and MMP14 in joint synovial tissue of RA (n=5) and OA (n=4) patients. p values were calculated by Mann–Whitney U test, *p < 0.05, **p < 0.01, ***p < 0.001.

Supplementary Figure 4. MiRNAs sequences and exosome identification. (a) 5 miRNAs sequences binding to HTR2A. (b-d) Nanoparticle tracking analysis (NTA), transmission electron microscopy (TEM, scale bars = 100 nm), and western blot (EV canonical positive markers including CD9, CD81, CD63, and negative marker Calnexin) were used to assess the quality of synovial fluid EV enrichments from OA (n=7) and RA (n=4) patients.
